# Precise Purity Analysis of High-Purity Lanthanum Oxide by Gravimetric Analysis Assisted With Trace Elemental Analysis by Inductively Coupled Plasma Mass Spectrometry

**DOI:** 10.3389/fchem.2022.888636

**Published:** 2022-07-15

**Authors:** Tsutomu Miura, Ayaka Wada

**Affiliations:** National Metrology Institute of Japan, National Institute of Advanced Industrial Science and Technology, Tsukuba, Japan

**Keywords:** gravimetric analysis, stoichiometry, ICP-MS, rare earth elements, lanthanum, traceability

## Abstract

Gravimetric analysis was used to determine the purity of high-purity La_2_O_3_ by stepwise conversions of the weighing forms. In this study, lanthanum in the sample was converted to La oxalate, La_2_O_3_, and La_2_(SO_4_)_3_ to evaluate the stoichiometry of the weighing forms for accurate gravimetric determination. The losses of La in the filtrate, the washing solution of the precipitate, and the mechanical loss of La during filtration were measured using inductively coupled plasma optical emission spectrometry. The weighing forms were evaluated by comparing the observed mass ratio with the theoretical value at each conversion step. The final converted La_2_(SO_4_)_3_ was consistent with the theoretical composition based on the observed mass ratio of La_2_(SO_4_)_3_/La_2_O_3_. Additionally, impurities in the high-purity La_2_O_3_ were determined by inductively coupled plasma tandem mass spectrometry. The purity of the original La_2_O_3_ sample was precisely determined to be 99.977 % ± 0.057% (mass fraction as La_2_O_3_; the value following “±” indicates the expanded uncertainty with a coverage factor *k* = 2.18) by a combination of the gravimetric analysis using the precipitation from the homogeneous solution method and verification of the weighing forms for La compound.

## Introduction

The traceability of inorganic analysis is established using high-purity inorganic compounds, the purity of which is accurately determined as primary standards (Vogl et al., 2018). The primary methods of measurement were used for accurate purity determination. The gravimetric analysis is one of the primary methods of measurement recognized by the Consultative Committee for Amount of Substance (CCQM) of the International Committee of Weights and Measures (CIPM) under the Metre Convention (Quin, 1997; Bièvre and Taylor 1997; Milton and Quinn 2001). The gravimetric analysis is a particularly important primary method of measurement in the purity assay analysis of chemical elements to which the coulometric analysis cannot be applied (Vetter et al., 1995; Hioki et al., 1990; Suzuki and Hioki 2006). In the gravimetric analysis, the analyte is selectively and quantitatively precipitated, and the mass of the precipitate or compound converted from the precipitate is weighed. The quantity of the analyte is directly determined by weighed mass. Establishing the exact mass balance of an analyte is essential for accurate and precise gravimetric analysis. Therefore, the weighing forms of the analyte should be evaluated based on stoichiometry. Additionally, the determination of the analyte is not in the weighed precipitate; that is, the mechanical losses (the residue on the beaker), and the quantity of the analyte in the filtrate and the washing solution, are required. However, few investigations have been carried out on the exact mass balance of the gravimetric analysis. For example, Vetter et al. (1995) demonstrated an improvement in its accuracy by combining a BaSO_4_ gravimetric analysis and an instrumental analytical method for the gravimetric determination of sulfate Vetter et al. (1995). Sulfate dissolved in the filtrate was determined as sulfur using inductively coupled plasma mass spectrometry (ICP-MS). Moreover, sodium and chloride ions occluded in the precipitate were measured using atomic absorption spectrometry and coulometric titration, respectively. Subsequently, the data resulting from the gravimetric analysis were corrected to enhance the accuracy of the gravimetric determination.

Rare earth elements are commonly measured in the fields of industry, geochemistry, and cosmochemistry (Yamamoto et al., 2011; Zhu et al., 2021; Ebihara et al., 2020); so there is a strong demand for the development of primary standard solution (Feng et al., 2019). In this study, gravimetric analysis was used to evaluate the purity of high-purity La_2_O_3_ as a raw material for a primary La standard solution based on the stoichiometric evaluation of the stepwise conversions of the weighed La compound. The weighing forms for La determination were evaluated by comparing the observed conversion mass ratios with the theoretical mass ratios for each combination of the weighing forms. First, lanthanum in the sample solution was precipitated as La oxalate by precipitation from a homogeneous solution method using diethyl oxalate (Gordon et al., 1959; Hagiwara 1956). The La-oxalate precipitate was converted by a stepwise method to La_2_O_3_ and La_2_(SO_4_)_3_ to evaluate the stoichiometry of the weighing forms. The quantity of La remaining in the filtrate and that dissolved in the solution used to wash the precipitate were determined using inductively coupled plasma optical emission spectrometry (ICP-OES). Finally, the purity of the high-purity La_2_O_3_ was determined from the analytical results of the evaluated weighing forms. Furthermore, the impurities in high-purity La_2_O_3_ were measured using inductively coupled plasma tandem mass spectrometry (ICP-MS/MS) to correct the gravimetric analytical results.

## Materials and Methods

### Instruments and Apparatus

A semi-micro balance (Mettler Toledo XP-205) was used to weigh the sample. The balance was calibrated using the Japan Calibration Service System (JCSS). The balances were installed in the balance room controlled at 25°C ± 2°C and 50% ± 10% relative humidity. All weighing was carried out in that balance room. A platinum crucible (nominal volume; 30 ml) was used as a container for ignition of the La-oxalate precipitate, followed by the gravimetric analysis using La_2_O_3_ and La_2_(SO_4_)_3_. Water was purified using a Merck Millipore Milli-Q Advantage A10 with a Q-Pod Element purification system. A pH meter (HM-30G, DKK-TOA Co. Tokyo, Japan) was used for pH measurements. An ICP-OES (PerkinElmer Optima 4300DV) was used to determine the unprecipitated La content in the filtrate and the washing solution of the La-oxalate precipitation. An ICP-MS/MS (iCAP TQ, Thermo Fisher Scientific, Bremen, Germany) was used to analyze the metal impurities in the original La_2_O_3_ sample.

### Reagents

Two hundred fifty grams of high-purity La_2_O_3_ (Alfa Aesar, REacton, powder, Lot No. U12A037, informative purity value: 99.999%) was purchased from FUJIFILM Wako Pure Chemicals Corporation (Osaka, Japan). Hydrochloric acid, HNO_3_, HF, and H_2_SO_4_ (Ultrapur-100 grade) were purchased from Kanto Chemicals Co. Inc. (Tokyo, Japan). A 50% diethyl oxalate solution was prepared by dissolving diethyl oxalate (JIS analytical grade, FUJIFILM Wako Pure Chemicals Corporation, Osaka, Japan) in ethanol (JIS analytical grade, FUJIFILM Wako Pure Chemicals Corporation, Osaka, Japan). A 2% oxalic acid solution was prepared by dissolving oxalic acid (JIS analytical grade, FUJIFILM Wako Pure Chemicals Corporation, Osaka, Japan) in water. A NIST SRM 3127a La standard solution was used to determine La in the filtrate, washing solution, and residues resulting from mechanical loss. A Co solution intended as an internal standard for La determination with ICP-OES was prepared by dilution of JCSS Co standard solution. The Co standard solution (Co: 1000 mg dm^−3^) was purchased from Kanto Chemicals Co. Inc. The NMIJ CRM standard solutions (3635-a Y), NIST SRM standard solutions (3148a Sc, 3110 Ce, 3142a Pr, 3135a Nd, 3147a Sm, 3117a Eu, 3118a Gd, 3157a Tb, 3115a Dy, 3123a Ho, 3116a Er, 3160a Tm, 3166a Yb, and 3130a Lu), and NIST traceable standard solutions of SPEX XSTC-331 (Th) were used for the impurity analysis using ICP-MS/MS.

### Measurement Procedure of Metallic Impurities by ICP-MS/MS

Approximately 1.5 g of La_2_O_3_ was weighed in a 30-ml quartz crucible for impurity analysis. The sample in the quartz crucible was ignited at 900°C for 2 h using an electric furnace and then cooled in a silica gel desiccator for 1 h in the balance room. Subsequently, 1.2 g of La_2_O_3_ was precisely weighed in a 200-ml borosilicate glass beaker. Twenty milliliters of HNO_3_ (1 + 2) was added to the sample in the beaker. The sample was heated at 120°C on a hot plate until La_2_O_3_ was completely dissolved. After complete dissolution, the solution was cooled down to ambient temperature and then diluted as a stock solution to 1 kg with 0.2 mol L^−1^ HNO_3_ in a high-density polyethylene bottle (mass fraction of La; *ca*. 1 g kg^−1^). Three sub-sample solutions (mass fraction of La of 10 mg kg^−1^) were prepared for the impurity analysis by diluting the stock solution. Impurities were measured by ICP-MS/MS using the calibration curve method. Calibration standard solutions were prepared with mass fractions of 0 ng kg^−1^, 50 ng kg^−1^, 100 ng kg^−1^, and 250 ng kg^−1^. Table 1 lists the operating conditions for ICP-MS/MS. The ICP-MS/MS instrument was tuned before measurement in accordance with the manufacturer’s recommendations.

**TABLE 1:** Instrumental conditions of Thermo Fisher iCAP TQ ICP-MS/MS for impurity analysis.

	ICP source operation parameters
RF power	1550 W
Ar plasma gas flow	16 L/min
Ar auxiliary gas flow	1.0 L/min
Nebulizer gas flow	1.12 L/min
Nebulizer	Borosilicate glass concentric nebulizer
Sample uptake rate	0.3 ml min^−1^
Spray chamber	Borosilicate glass cyclonic spray chamber
Sampling cone and skimmer cone	Ni sampling cone, Ni skimmer cone
	Spectrometer operating parameters
Measured isotopes and measurement modes	Single Quadrupole Mass Spectrometry mode
	^89^Y, ^141^Pr, ^142^Ce, ^143^Nd, ^145^Nd, ^146^Nd, ^147^Sm, ^149^Sm, ^151^Eu, ^157^Gd, ^159^Tb, ^161^Dy, ^163^Dy, ^165^Ho, ^166^Er, ^167^Er, ^169^Tm, ^172^Yb, ^175^Lu, ^232^Th
	H_2_ gas collision reaction mode; H_2_ gas flow 10 ml min^−1^
	^45^Sc (45; m/z at QMS1, 45: m/z at QMS2)
Dwell time	100 ms
Number of sweeps	Ten times
Measurement repetition	Five times

### Analytical Procedures of Gravimetric Analysis

**FIGURE 1:**
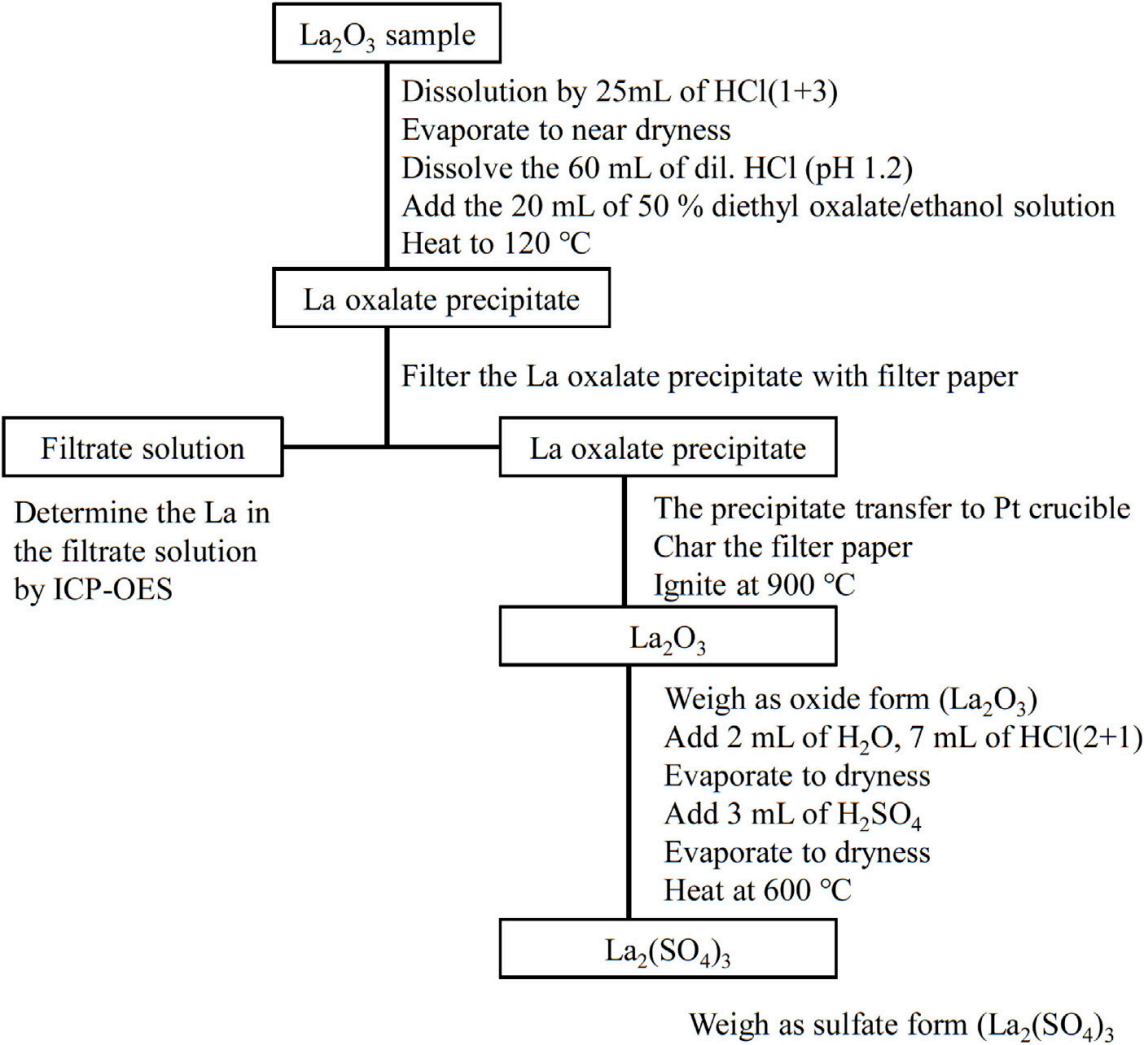
Schematic diagram of the gravimetric analysis procedure for La.

The gravimetric analysis procedure comprises three steps: 1) sampling and separation of La using La-oxalate precipitation from homogeneous solution, 2) the gravimetric analysis using La_2_O_3_, and 3) conversion of the oxide to sulfate and weighing as the product, La_2_(SO_4_)_3_. A description of the steps is provided in the following text. The buoyancy effect during weighing was corrected. The schematic diagram of the gravimetric procedure is shown in Figure 1.

### Sampling and Separation of La Using La-Oxalate Precipitation From Homogenous Solution

Before the gravimetric analysis, La_2_O_3_ samples in the quartz crucible were ignited at 900°C for 2 h and then allowed to cool in a silica gel desiccator for 1 h in the balance room. The mass of La_2_O_3_ samples was approximately 96.5% of the original sample mass in this pre-ignition procedure. After the preignition process, 10 samples of La_2_O_3_ of approximately 1 g each were weighed precisely in 200 ml borosilicate glass beakers. Twenty-five milliliters of HCl (1 + 3) was added to the beaker covered with a watch glass. The beaker was heated at 120°C on a hot plate until the La_2_O_3_ sample was completely dissolved, after which the solution was cooled to ambient temperature. The watch glass was removed and the solution was gently heated until it evaporated to near dryness. After evaporation, the beaker was cooled to ambient temperature. Then, 60 ml of diluted HCl solution adjusted to pH 1.2 by NH_4_OH was added to the sample. The sample solution was heated at 120°C on a hot plate until it was completely dissolved. After cooling to ambient temperature, 20 ml of the 50% diethyl oxalate/ethanol solution was added to the sample solution. The sample solution was heated overnight at 120°C on a hot plate for La oxalate precipitation from a homogeneous solution. Diethyl oxalate was slowly producing oxalate anions by heating (Gordon et al., 1959; Hagiwara 1956).

$${\left(C_{2}H_{5}\right)}_{2}C_{2}O_{4}+2H_{2}O\to 2C_{2}H_{5}OH+2H^{+}+C_{2}O_{4}^{2-}$$

After cooling to ambient temperature, the La oxalate precipitate was filtered with a filter paper (No. 5C, diameter; 9 cm, ADVANTEC TOYO, Tokyo, Japan) and washed with water. The filtrate was collected in a 200-ml borosilicate glass beaker. A small amount of the precipitate remaining in the beaker was transferred onto the filter paper with a 2% oxalic acid solution. The La oxalate precipitate on the filter paper was washed with 5 ml of the 2% oxalic acid solution. The filtrate volume was approximately 100 ml at the completion of filtration. The filtered precipitate was then transferred to a 30 ml Pt crucible. After charring the filter paper, the precipitate was ignited at 900°C for 1 h in an electric furnace, allowed to cool for 1 h in a silica gel desiccator in the balance room, and weighed until the mass remained constant. The La oxalate precipitate was converted to the oxide form, La_2_O_3_, by this ignition process. The sequence of drying, cooling, and weighing was repeated until the mass remained constant. In this study, the criterion used for a constant mass was a variation of less than 0.1 mg.

### Conversion From La_2_O_3_ to La_2_(SO_4_)_3_

After a constant mass was reached, the oxide in the Pt crucible was dissolved in 2 ml of H_2_O and 7 ml of HCl (2 + 1). After the dissolution of the oxide, the solution in the Pt crucible was placed on a hot plate and heated until it became dry. Three milliliters of H_2_SO_4_ (1 + 1) were added to the Pt crucible to convert the sulfate from the oxide. The Pt crucible was then heated at 350°C on a hot plate until the excess H_2_SO_4_ completely evaporated. After evaporation, the Pt crucible was transferred to an electric furnace. The temperature of the electric furnace was increased stepwise to 450°C for 2 h and 550°C for 4 h. Finally, the sample was heated at 600°C for 1 h to complete the conversion to La_2_(SO_4_)_3_. La_2_(SO_4_)_3_ was cooled in a P_2_O_5_ desiccator in the balance room and then weighed. The sequence of heating at 600°C, cooling, and weighing was repeated until constant mass was attained. Thus, in this study, La_2_(SO_4_)_3_, which was converted from La_2_O_3_ by igniting the La oxalate precipitate, was heated to 600°C to ensure that volatile compounds were eliminated.

### The Measurement of Unprecipitated La by ICP-OES

Unprecipitated La in the filtrate and washing solution was measured using PerkinElmer Optima 4300DV ICP-OES to evaluate the loss of La. The measurement conditions for ICP-OES are listed in Table 2. The analytical results were calculated from the mass of La_3_O_3_, La_2_(SO_4_)_3_, and the amount of unprecipitated La in the filtrate, remaining in the washing solution.

**TABLE 2:** Instrumental conditions of PerkinElmer Optima 4300DV ICP-OES.

	ICP source operation parameters
RF power	1300 W
Plasma gas flow	Ar 15 L min^−1^
Auxiliary gas flow	Ar 0.2 L min^−1^
Nebulizer gas flow	Ar 0.7 L min^−1^
Nebulizer	Borosilicate glass conical nebulizer
Spray chamber	Borosilicate glass cyclonic spray chamber
Sample uptake	1 ml min^−1^
	Spectrometer operating parameters
Viewing direction	Axial
Signal measurement mode	Peak area (3 point)
Background correction	Manually selected, 2 point interpolations
Measurement time	Auto
Measurement replications	Ten times
Measured emission line	La: 407.735 nm, Co: 238.892 nm

## Results and Discussion

### Analytical Results of Impurities

Sixteen elements that possibly coprecipitated on La-oxalate at pH 1.2 solution were measured in the original La_2_O_3_ sample by ICP-MS/MS. The precipitation reaction of oxalate in acidic solution at pH 1.2 is selective to rare earth elements and Th (IV). The other elements, such as Fe, Al, Ca, Sr, and Na, form soluble oxalate and are not precipitated in acidic solution at pH 1.2 [Woyski et al., 1964, Hagiwara 1956; Miura and Isogai 2017]. The results of the impurity analysis using ICP-MS/MS are shown in Table 3. The elements for which no quantitative value could be obtained showed an upper limit. In this case, the standard uncertainty was calculated by dividing the upper limit by 2√3. The total mass fraction of impurities in the original La_2_O_3_ sample was calculated by summing the measured quantitative values and half values of the upper limit for the elements for which the upper limit was obtained. The obtained total mass fraction of impurities was 1.0 mg kg^−1^±0.19 mg kg^−1^ (*u*; combined standard uncertainty, *k* = 1).

**TABLE 3:** Analytical results of impurities in the original La_2_O_3_ sample.

Element	Measured mass fraction (mg kg^−1^)	Relative standard uncertainty due to standard solution (%)	Relative standard uncertainty due to calibration (%)	Relative standard uncertainty due to measurement repeatability (%)	Combined standard uncertainty (mg kg^−1^)
Sc	<0.2				0.057
Y	0.20	0.05	2.3	1.9	0.0059
Ce	0.09	0.11	2.6	2.8	0.0035
Pr	0.048	0.11	2.0	1.6	0.0012
Nd	0.13	0.12	2.5	2.1	0.0043
Sm	<0.011				0.0032
Eu	<0.002				0.00057
Gd	<0.010				0.0029
Tb	0.0032	0.14	5.9	2.4	0.0002
Dy	0.017	0.11	6.9	4.9	0.0014
Ho	0.018	0.11	2.2	2.6	0.0006
Er	0.033	0.12	2.6	4.5	0.0017
Tm	0.01	0.18	1.9	4.6	0.0019
Yb	<0.62				0.18
Lu	0.0039	0.14	4.4	4.2	0.0002
Th	<0.0022				0.001
Total amounts of impurities	1.0				0.19

### Selection of Precipitation Reaction

The selection of the precipitation reaction and weighing form is of fundamental importance for a precise gravimetric analysis. Lanthanum fluoride, La-hydroxide, La oxalate, and La-8-quinolinolate complex can also be used for the precipitation of La (Woyski and Harris, 1964). Lanthanum oxalate is a relatively selective precipitate in the aforementioned precipitation reactions because it can be precipitated in a lower pH solution (Hagiwara 1956; Miura and Isogai 2017). Furthermore, the oxalate precipitate obtained using the precipitation from the homogeneous solution method (PFHS method) can produce a crystalline precipitate with a small amount of coprecipitate. In this study, La oxalate was precipitated at pH 1.2 with diethyl oxalate using the PFHS method. After filtration of the La oxalate precipitate, the precipitate was ignited to convert oxide. Then oxide was converted to sulfate for stoichiometric evaluation of the weighing form.

### Determination of the Purity of the Original La_2_O_3_ Sample Based on the Stoichiometric Evaluation

The results of the gravimetric analysis are presented in Tables 4, 5. The results were corrected by considering the amounts of La remaining in the filtrate and washing solution of the precipitate. In addition, the purity value obtained was corrected by subtracting the total mass fraction of impurities to remove their influence. The combined standard uncertainty of purity was estimated from the sensitivity and linearity of the balance, the repeatability of weighing the oxide form as La_2_O_3_, and the ICP-OES determination of unprecipitated La and impurities. However, the uncertainty related to the evaluation of stoichiometry of the weighing form was not taken into consideration.

**TABLE 4:** Analytical results for purity of the original La_2_O_3_ sample by the oxide weighing method^a^ ^,^ ^b^.

Run no.	Sample mass, g	Recovered La_2_O_3_, g	Unprecipitated La, μg	La in precipitate, %	Unprecipitated La, %	Purity of original La_2_O_3_ ^c^, %
1	0.94130	0.94062	386 ± 4.1	99.928	0.048	99.976 ± 0.003
2	0.95886	0.95874	525 ± 4.8	99.988	0.064	100.052 ± 0.009
3	0.77350	0.77274	474 ± 4.2	99.901	0.072	99.973 ± 0.005
4	0.87258	0.87149	467 ± 4.2	99.875	0.063	99.938 ± 0.003
5	0.99128	0.99056	370 ± 3.6	99.928	0.044	99.972 ± 0.005
6	0.87286	0.87206	515 ± 4.0	99.908	0.069	99.977 ± 0.006
7	0.86632	0.86570	492 ± 4.6	99.928	0.067	99.995 ± 0.005
8	0.88910	0.88874	420 ± 3.4	99.959	0.055	100.014 ± 0.011
9	0.85972	0.85850	553 ± 3.5	99.859	0.075	99.934 ± 0.006
10	0.98191	0.98119	532 ± 3.8	99.926	0.064	99.990 ± 0.005
					Mean	99.982 ± 0.012

a Calculated based on the weighing form as La_2_O_3_.

b Each value following "±" indicates the combined standard uncertainty (*k* = 1).

c The purity value of original La_2_O_3_ was corrected by the mass fraction of impurities.

**TABLE 5:** Analytical results for purity of original La_2_O_3_ by the La_2_(SO_4_)_3_ conversion method^a^ ^,^ ^b^.

Run no.	Weighed La_2_(SO_4_)_3_, g	Purity of original La_2_O_3_ ^c^, %	Mass ratios of La_2_(SO_4_)_3_/La_2_O_3_ found/theoretical^d^
1	1.63359	99.947 ± 0.008	0.99970
2	1.66432	99.978 ± 0.006	0.99926
3	1.34344	100.049 ±0.007	1.00075
4	1.51412	99.948 ±0.008	1.00010
5	1.72069	99.963 ±0.006	0.99991
6	1.51479	99.965 ±0.013	0.99988
7	1.50312	99.942 ± 0.007	0.99947
8	1.54332	99.974 ± 0.006	0.99960
9	1.49184	99.962 ±0.006	1.00029
10	1.70475	100.001 ± 0.008	1.00012
	Mean	99.973 ± 0.013	0.99991

a Calculated based on the weighing form as La_2_(SO_4_)_3_.

b Each value following "±" indicates combined standard uncertainty (*k* = 1).

c The purity value of original La_2_O_3_ was corrected by the mass fraction of impurities.

d The theoretical ratio of La_2_(SO_4_)_3_/La_2_O_3_ = 1.7372.

Table 4 shows the results of the gravimetric analysis using the oxide form of La_2_O_3_. The quantities of unprecipitated La are also listed in the table. The quantity of unprecipitated La ranged from 0.037 % to 0.064% under these conditions. This indicates that the PFHS method using diethyl oxalate is quantitatively precipitated La-oxalate in a pH 1.2 acidic solution. It was revealed that 99.9% or more of La could be precipitated by the PFHS method using diethyl oxalate. As is clear from Table 4, the purity of the original La_2_O_3_ sample was measured to 99.982 % ± 0.012% (*u*; combined standard uncertainty, *k* = 1) by the gravimetric analysis using the oxide form as La_2_O_3_.

Table 5 presents the results of the gravimetric analysis using the sulfate form, La_2_(SO_4_)_3_. The combined standard uncertainty of the purity was estimated from the sensitivity and linearity of the balance, the repeatability of weighing the sulfate form as La_2_(SO_4_)_3_, and the ICP-OES determination of unprecipitated La and impurities. However, the uncertainty related to the evaluation of stoichiometry of the weighing form was not taken into consideration.

According to the results, the purity of the original La_2_O_3_ sample was measured as 99.973 % ± 0.013% (*u*; combined standard uncertainty, *k* = 1). This purity value is consistent with the results obtained by the gravimetric analysis of the oxide form as La_2_O_3_ (Table 4). Furthermore, the mass ratios of La_2_(SO_4_)_3_/La_2_O_3_ in Table 5 are close to the theoretical value; the mean of the mass ratio of La_2_(SO_4_)_3_/La_2_O_3_ to the theoretical value is 0.99991. These facts validated that the compositions of converted La_2_(SO_4_)_3_ and La_2_O_3_ were close to the theoretical values. Therefore, the purity values of the original La_2_O_3_ sample could be determined by the arithmetic mean of the value obtained from the gravimetric analysis using the oxide form as La_2_O_3_ and the gravimetric analysis using the sulfate form as La_2_(SO_4_)_3._

### Calculation of Measurement Uncertainty

The measurement uncertainties derived from this method are listed in Table 6. In this study, the reproducibility of the gravimetric analysis using the oxide form of La_2_O_3_ (Table 4) and the gravimetric analysis using the sulfate form of La_2_(SO_4_)_3_ (Table 5), as well as the uncertainty of the stoichiometry of the weighing forms, the uncertainty of weighing the original La_2_O_3_ sample, the uncertainty in the impurity analysis, and the uncertainty in molecular weights of La_2_O_3_, and La_2_(SO_4_)_3_ were considered as uncertainty components. The uncertainties based on the reproducibility of the gravimetric analysis using the oxide form as La_2_O_3_ (Table 4) and the gravimetric analysis using the sulfate form as La_2_(SO_4_)_3_ (Table 5) were determined by calculating the relative standard deviation of the mean values of the gravimetric analysis results and number of samples. The uncertainty of the stoichiometry of the weighing form was calculated from the mean of the absolute value of the difference between the analytical values by the gravimetric analysis using the oxide form as La_2_O_3_ and the gravimetric analysis using the sulfate form as La_2_(SO_4_)_3_. The uncertainty regarding the stoichiometry of the weighing forms was calculated by the root mean square of the deviation for each sample obtained by considering the difference between the purity values by the two weighing forms of each sample as a rectangular distribution and dividing it by √3 . The uncertainties regarding the reproducibility of the gravimetric analysis and the stoichiometry of weighing forms are shown in Table 6.

**TABLE 6:** Uncertainties about the reproducibility of the gravimetric analysis and the stoichiometry of weighing forms.

Run no.	Purity of original La_2_O_3_ by oxide form, %	Purity of original La_2_O_3_ by sulfate form, %	Mean of the purity values^a^, %	Absolute difference of the purity values^b^, %	$\left|\text{Dif}.\right|/\sqrt{3}$ , %
1	99.976 ± 0.003	99.947 ± 0.008	99.961	0.030	0.017
2	100.052 ±0.009	99.978 ± 0.006	100.015	0.074	0.043
3	99.973 ± 0.005	100.049 ± 0.007	100.011	0.075	0.043
4	99.938 ± 0.003	99.948 ± 0.008	99.943	0.010	0.0058
5	99.972 ± 0.005	99.963 ± 0.006	99.967	0.0085	0.0049
6	99.977 ± 0.006	99.965 ± 0.013	99.971	0.012	0.0069
7	99.995 ± 0.005	99.942 ± 0.007	99.968	0.053	0.030
8	100.014 ± 0.011	99.974 ± 0.006	99.994	0.040	0.023
9	99.934 ±0.006	99.962 ± 0.006	99.948	0.029	0.016
10	99.990 ± 0.005	100.001 ± 0.008	99.996	0.012	0.0069
		Mean	99.977	Root mean square	0.024^c^
		RSD	0.025		
		$\text{RSD}/\sqrt{n}$	0.0080^d^		

a Mean of the purity values obtained in the two weighing forms.

b Absolute difference in purity values obtained from two weighing forms.

c The root mean square was used as uncertainty for the stoichiometry of weighing forms.

d The $\text{RSD}/\sqrt{n}$ was used as uncertainty of the reproducibility of the gravimetric analysis.

The calculation results of measurement uncertainties are listed in Table 7, where the main component of the combined standard uncertainty is the uncertainty of stoichiometry of the weighing form in the gravimetric analysis. The effective degree of freedom calculated by the Welch-Satterthwaite equation was 12. The relative expanded uncertainty of the purity value of lanthanum oxide was evaluated to be 0.057% using coverage factor *k* = 2.18, which represents a 95% confidence interval with the *t*-distribution from the calculated effective degrees of freedom.

**TABLE 7:** Contribution of measurement uncertainty in the purity analysis of the original La_2_O_3_ sample.

Uncertainty component	Relative standard uncertainty, %
Repeatability of the gravimetric analysis	0.0080
Stoichiometry of weighing forms	0.024
Weighing for original La_2_O_3_	0.00066
Weighing for oxide form	0.00069
Weighing for sulfate form	0.00069
Impurities	0.000019
Molecular weights	
La_2_O_3_	0.00040
La_2_(SO_4_)_3_	0.0053
Combined standard uncertainty	0.026
Coverage factor	2.18
Expanded uncertainty(*U*), relative (%)	0.057

## Conclusion

A gravimetric analysis method using a stepwise conversion of weighing forms was used to determine the purity of the high-purity La_2_O_3_ sample. Lanthanum in the sample was converted stepwise to La oxalate, La_2_O_3_, and La_2_(SO_4_)_3_ to evaluate the accuracy of the weighing forms. In addition, the impurities in the original La_2_O_3_ sample, that is, possible coprecipitate on La oxalate, were determined by ICP-MS/MS. The observed mass ratios of the weighed forms were compared to the corresponding theoretical values. The composition of converted La_2_(SO_4_)_3_ was close to the theoretical value because the mass ratio of observed La_2_(SO_4_)_3_/La_2_O_3_ to the theoretical value was close to unity (0.99991). The purity of the original La_2_O_3_ sample was precisely determined to be 99.977 % ± 0.057% (expanded uncertainty, *k* = 2.18) using the gravimetric analysis including stoichiometric evaluation.

## Data Availability

The original contributions presented in the study are included in the article/Supplementary Material; further inquiries can be directed to the corresponding author.
